# ^1^H NMR Sensor for Nondestructive Characterization of Organic and Inorganic Materials

**DOI:** 10.3390/s24237692

**Published:** 2024-11-30

**Authors:** Floriberto Díaz-Díaz, Prisciliano F. de J. Cano-Barrita, Frank M. León-Martínez, Víktor Acevedo-Arzola

**Affiliations:** 1 Instituto Politécnico Nacional/CIIDIR Unidad Oaxaca, Calle Hornos No. 1003, Col. Noche Buena, Santa Cruz Xoxocotlán, Oaxaca 71230, Mexico; fdiazd@ipn.mx (F.D.-D.); fmleonm@ipn.mx (F.M.L.-M.); 2 Conahcyt-Instituto Politécnico Nacional/CIIDIR Unidad Oaxaca, Calle Hornos No. 1003, Col. Noche Buena, Santa Cruz Xoxocotlán, Oaxaca 71230, Mexico; 3 School of Engineering and Architecture, Universidad La Salle Oaxaca, Camino a San Agustín No. 407, Santa Cruz Xoxocotlán, Oaxaca 71230, Mexico; 014405021@ulsaoaxaca.edu.mx

**Keywords:** relaxometry, nondestructive testing, profile

## Abstract

Nuclear magnetic resonance relaxation of the proton spins of liquid molecules and their evolution during processes such as drying, fluid flow, and phase change of a sample can be monitored in a nondestructive way. A unilateral ^1^H NMR sensor made with a permanent magnet array, inspired by the NMR MOUSE, with an RF coil tuned to 11.71 MHz was developed. This creates a sensitive homogeneous measuring volume parallel to the sensor surface and located 14 mm from its surface, allowing contactless measurements from the sample’s interior. As this sensitive volume is moved across the sample using a semi-automatic linear displacement mechanism with millimetric precision, spatial T_2_ lifetime and signal intensity 1D profiles can be obtained. To characterize the sensor’s sensitive volume, eraser samples were used. To evaluate the sensor’s ability to characterize different materials, cement paste samples containing ordinary and white Portland cement were prepared and measured at seven days of age. In addition, measurements were made on organic samples such as a Hass avocado and beef steak. Based on the results, a 1 mm spatial resolution of the sensor was achieved. The sensor was able to detect differences in T_2_ lifetimes in eraser specimens composed of layers of three different erasers. Also, a clear difference in T_2_ lifetimes and signal intensities was observed in cement pastes composed of white and ordinary Portland cement. On the other hand, it was possible to obtain signals from the peel and pulp of the avocado fruit, as well as from the fat and meat in a beef steak in a nondestructive way. The T_2_ lifetimes of the different materials agreed with those obtained using a commercial NMR spectrometer.

## 1. Introduction

Nuclear magnetic resonance (NMR) is a nondestructive and non-invasive technique that enables the observation of changes in the microstructure of materials without the need for sample extraction or causing damage to the object of study. This technique is based on the absorption and emission of electromagnetic radiation by the nuclei of specific atoms subjected to a strong magnetic field and excited by radiofrequency pulses [1,2,3]. By measuring the NMR signal, information about the structure and composition of the sample is obtained.

In the context of cement-based materials, NMR has been used to study hydration [4], moisture content, pore refinement, compressive strength [5], carbonation depth [6], and moisture content profiles during capillary water absorption [7]. In the agri-food industry, NMR has also proven to be a valuable tool. For example, NMR spectroscopy has been used to detect adulterations in vegetable oils, determine the composition of fatty acids, the presence of trans fats, and other parameters important for quality and health [8], as well as in the identification of contaminants, pesticide residues, additives, and other unwanted compounds in food [9].

Relaxometry, another NMR tool, allows the study of food components through relaxation times and signal intensity [10]. In this context, unilateral NMR emerges as a promising technique derived from traditional NMR, allowing the application of these relaxometry principles to study a specific region of the sample without the need to place the entire object within a homogeneous magnetic field. This technique benefits from low-field unilateral sensors, which are mainly classified into two types: those that generate a gradient B_0_ magnetic field and those that create a B_0_ magnetic field sweet spot [11].

Sensors that generate a gradient B_0_ magnetic field are used to obtain spatial information about the interior of the sample, which allows the creation of depth profiles of the studied material. The simplest design of this type is the single bar magnet, which produces a B_0_ field perpendicular to its surface [12]. For its operation, a coil is required that generates a transverse B_1_ field, for which a “figure 8” coil is generally used. However, the low homogeneity of this coil limits the penetration of the field into the sample [11], which presents difficulties in applications that demand exploring deeper volumes or require a more uniform excitation. One of the most used geometries is the “U” shaped magnet, which generates a B_0_ field parallel to the surface. The first version of the NMR MOUSE [13] was developed with this geometry, consisting of two semicylindrical shape magnets separated by a gap on an iron plate. This sensor allowed profiles to be obtained by varying the frequency according to the desired depth. Subsequently, improvements were introduced to the design, which mainly consisted of dividing the U-shaped magnet into two parts, introducing a small additional gap between these divisions to adjust the uniformity of the magnetic field laterally [14,15]. These improvements made the NMR MOUSE evolve into an advanced sensor capable of making profiles with microscopic resolution.

On the other hand, sensors that generate an optimal magnetic field point B_0_ are used to maximize the size of the sensitive volume and obtain homogeneous measurements without considering spatial information. Such sensors have proven helpful in agri-food and industrial applications, such as assessing intramuscular fat content in live cattle [16], quantifying fat and water in fresh tuna meat [17,18], and measuring moisture in low-grade pulverized coal [19]. Among the most representative examples of this category are the barrel magnet [20], the NMR MOLE [21], and the three-magnet array [22,23].

The aim of the present work was to develop a unilateral NMR sensor, based on the NMR MOUSE [14], that allows measurements from the surface to the interior of the sample with a millimetric resolution. This allows a significant cost reduction when measuring samples that do not require microscopic resolution like the NMR MOUSE. A design based on an optimal point would not be suitable since it generates a sensitive volume that is too large, which would reduce the resolution and make it difficult to obtain profiles. Therefore, the design will be based on a modified U-shaped geometry, which will allow adequate control of the magnetic field, ensuring that the sensitive area is far from the surface, allowing the use of more efficient antennas such as the surface spiral type and the possibility of obtaining profiles with sufficient resolution without compromising cost. In addition, semi-automation of measurements is incorporated, which will allow the sensor to make horizontal and vertical millimetric movements.

## 2. Materials and Methods

### 2.1. Sensor Design and Construction

**Figure 1:**
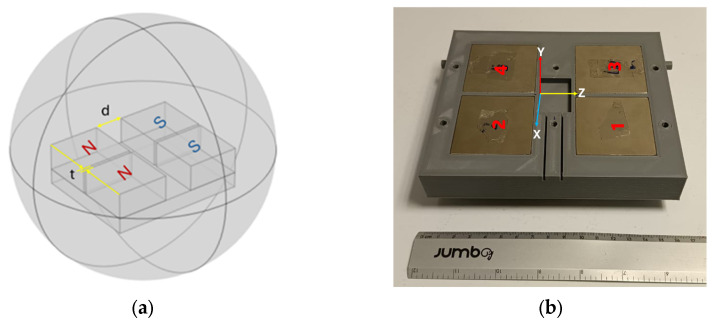
(**a**) Configuration of the magnet array optimized by simulation, where the distances d and t were 28 mm and 3 mm respectively, (**b**) Constructed magnet array, the magnets were placed inside a 3D-printed case to keep them in place according to the simulation. The numbers in red refer to each specific magnet whose magnetic field was measured.

The unilateral NMR sensor mainly consists of an array of NdFeB permanent magnets and a radiofrequency antenna. Four N35 grade NdFeB rectangular magnets (Magnetika Saiffe, Guadalajara, Mexico) measuring 50 mm × 50 mm × 25 mm were used. The magnetic field strengths on their surfaces were 368 mT, 377 mT, 370 mT, and 377 mT for magnets 1, 2, 3, and 4, respectively, which were measured using a gaussmeter HT-20 (Hangzhou Best Magnet Co. Ltd., Hangzhou, China). The COMSOL Multiphysics^®^ version 3.4 finite element software (COMSOL, Burlington, MA, USA) was used to perform static magnetic field simulations of the magnet array based on a modified U-shaped geometry by varying the separation distance d and t between the magnets (Figure 1a). To achieve the best configuration that permitted reaching a homogeneous volume of the magnetic field away from the surface, like the NMR MOUSE magnet array [14,15,24]. Based on the simulation results, the array was constructed by placing the magnets on a mild steel plate measuring 105 mm × 130 mm × 15 mm, arranged as shown in Figure 1a. A 3D-printed case was used to hold the magnets in place (Figure 1b). Because the four magnets were not perfectly matched in the magnetic field strength across their surface, it was demonstrated both in simulation and construction to place the magnets in the order shown in Figure 1b.

**Figure 2:**
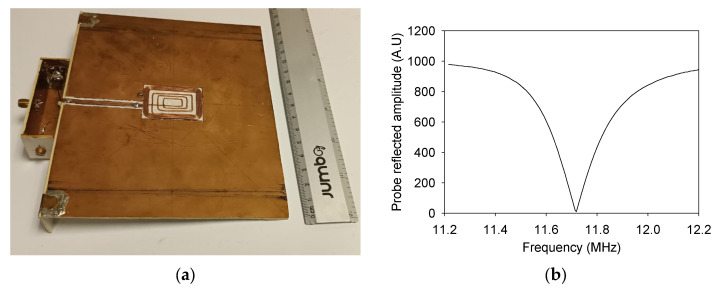
(**a**) A photograph of the constructed RF antenna and (**b**) Reflected signal from the transmitter as a function of frequency, where the minimum value indicates the optimal tuning corresponding to 11.71 MHz.

Subsequently, a surface RF antenna was designed, constructed, and tuned to a frequency of 11.71 MHz for ^1^H resonance frequency, according to the Larmor Equation (1), using fixed and variable non-magnetic capacitors. A rectangular spiral antenna measuring 20 mm × 30 mm (Figure 2a), was constructed with 21 AWG copper wire and coated with epoxy resin. It comprises ten turns, with a bandwidth ∆f = 100.5 kHz and a quality factor Q = 116. The S11 parameter (scattering parameter) was evaluated to ensure proper antenna matching to the system and minimize reflected power. This parameter measures the level of reflected energy at the antenna input port; a low S11 indicates that the antenna is well-matched to the system, allowing most of the power to be transferred without significant losses. In the case of the built antenna, the minimum of S11 occurs at the frequency of 11.71 MHz (Figure 2b), confirming proper matching for signal transmission at this frequency. The antenna was installed on a 1.5 mm-thick printed circuit board, covering the magnet array’s surface to minimize the effects of eddy currents. When combined, the antenna and the board have a total thickness of 2.9 mm, reducing the available measurement distance to 11.1 mm. This distance represents the space between the antenna surface and the magnetic field’s homogeneous zone.

(1) $$\omega =\gamma *B_{0}$$

where ω is the Larmor frequency in MHz, γ is the gyromagnetic ratio in MHz/T, and B_0_ is the strength of the static magnetic field in T.

### 2.2. Linear Movement Mechanism for Sensor Displacement

**Figure 3:**
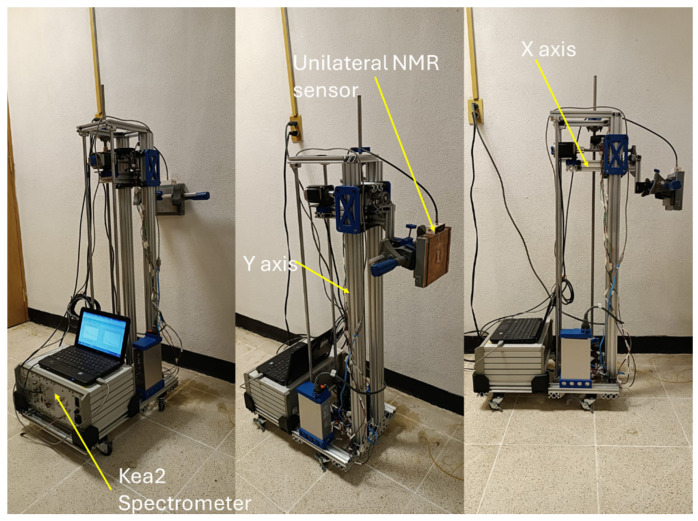
Photograph of automatic mechanism for displacement of the unilateral NMR sensor.

A semi-automatic CNC displacement mechanism was designed and built to carry out NMR measurements at precise sample positions. The mechanism is 110 cm tall and has a base measuring 53 cm × 35 cm (Figure 3). It utilizes structural aluminum profiles of various cross-sectional dimensions (40 mm × 40 mm, 40 mm × 20 mm, and 20 mm × 20 mm) to ensure strength and rigidity. Additionally, it incorporates smooth 12-mm diameter stainless steel rods, a 16-mm diameter spindle with corresponding nuts, and various parts manufactured via 3D printing.

The mechanism comprises two primary axes: the longitudinal *X*-axis and the vertical *Y*-axis. The *X*-axis controls longitudinal movement, allowing the sensor to approach or retract from the sample’s surface within a maximum displacement of 10 cm. The *Y*-axis permits the vertical movement of the sensor, with a maximum displacement of 75 cm. These displacements are driven by NEMA 23 OK57H18112A 4.2A 3 Nm (Oukeda Electric Appliance Co., Ltd., Changzhou, China) stepper motors and drivers DM556 (Leadshine Technology Co., Ltd., Shenzhen, China), which offer precise and reliable positioning control. The movement instructions are transmitted to the motors from an ESP32-WROOM-32U (Espressif Systems, Shanghai, China) development board, which manages and coordinates axis movements along with controlling other system functions.

### 2.3. NMR Measurements

**Figure 4:**
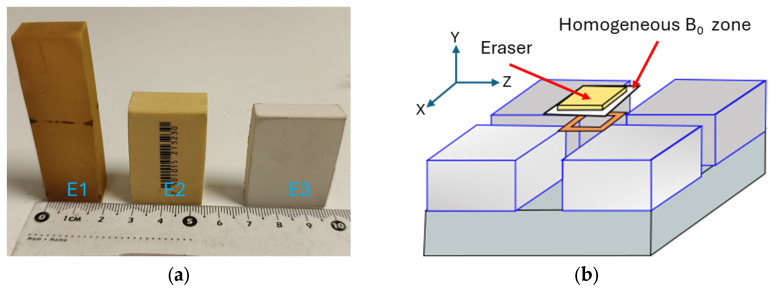
Materials used in sensor characterization. (**a**) Samples of three types of erasers. (**b**) The 2 mm thick eraser slice was moved to different positions within the homogeneous magnetic field zone B_0_ along the *Y*-axis.

To characterize the sensor, we used different types of erasers for the NMR measurements (Figure 4a). These materials were used to conduct tests to determine parameters for the CPMG technique, such as the amplitude and the 90° and 180° pulse widths.

We measured the thickness of the region excited by the RF antenna using a 2-mm-thick slice of an eraser. The eraser was displaced in 0.2 mm increments upward and downward along the vertical axis of the homogeneous zone of the magnetic field generated by the magnet array (Figure 4b). An NMR signal was acquired at each position using the CPMG technique [25]. Based on the signal intensity, two profiles were obtained to determine the thickness of the region that can be excited by the sensor antenna. To establish the sensor’s upper and lower sensitive limits, we identified the position where the signal intensity became relatively constant in the downward profile, aligning with the last position where the signal intensity was still zero in the upward profile. The upper limit was determined by finding the position where the signal intensity became relatively constant in the upward profile and coincided with the last position where the signal intensity was still zero in the downward profile.

**Figure 5:**
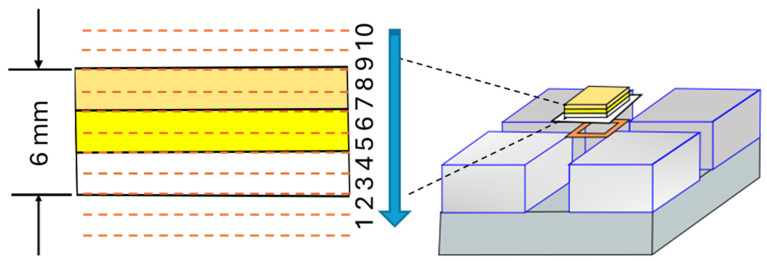
On the left, a representation of the specimen made with layers of three different types of erasers is shown. The dashed red lines indicate the positions where signal measurements were taken, with the sensor moved in 1 mm steps from top to bottom, as indicated by the blue arrow.

Subsequently, an eraser sample composed of the three layers of each type of eraser was made. Each eraser layer measured 2 mm × 23.6 mm × 19.6 mm, resulting in a total thickness of 6 mm (Figure 5). The specimen was then measured in 10 vertical positions by moving the sensor, which consequently moved the sensitive volume from top to bottom in 1 mm steps. At each position, a CPMG NMR signal acquisition was performed.

### 2.4. Testing the Applicability of the Sensor

Two cylindrical (∅ = 4 cm and h = 4 cm) specimens of white Portland cement (WPC) and ordinary Portland cement (OPC) at a water-to-cement ratio (w/c) of 0.60 were prepared. Also, one additional WPC sample was prepared with a w/c ratio of 0.70. These were measured with the CPMG technique at the age of 14 days.

**Figure 6:**
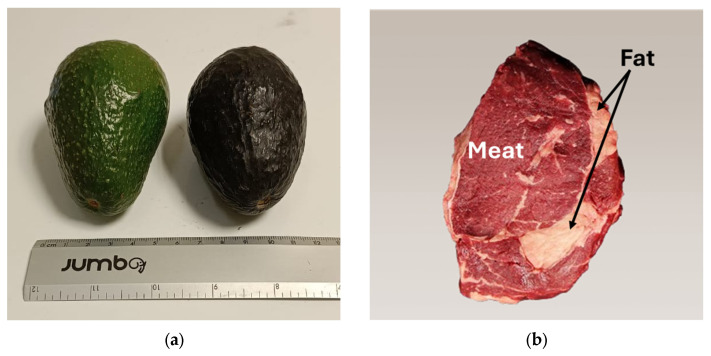
Organic material for the NMR measurements with the sensor (**a**) Hass avocado samples and (**b**) Beef steak.

For organic material, measurements were performed on Hass avocado samples at their final ripening stage and on beef steak, as shown in Figure 6. NMR measurements using the unilateral sensor were performed on the avocado’s pulp and skin without cutting. Then the pulp and skin were extracted and measured separately using a Maran DRX HF 12/50 system (Oxford Instruments Ltd., Abingdon, Oxford, UK) operating at 12.9 MHz. These last measurements were used to compare with those obtained using the sensor.

For the beef steak, measurements with the unilateral sensor were performed separately on the part containing only fat and on the part containing only meat.

All NMR experiments with the sensor were performed using a Kea^2^ spectrometer (Magritek Limited, Wellington, New Zealand). Table 1 provides the parameters for the CPMG NMR measurements using the unilateral sensor and the Maran DRX HF 12/50 instrument for the different materials.

For each material analyzed, the T_2_ values and the amplitude associated with each component were determined by fitting the CPMG echo train to the exponential decay model described by Equation (2).

(2) St=∑i=1nAie−tT2,i

where S(t) is the NMR signal measured as a function of time t, A_i_ is the amplitude associated with component i. This term reflects the relative contribution of that component to the total signal and is related to the number of protons in that population. e^−t/T2,1^ is the exponential term describing the signal decay associated with component i. T_2,i_ is the transverse relaxation time for component i. This parameter indicates how quickly magnetization is lost due to interactions between protons and their environment. n is the number of components detected in the signal decay.

## 3. Results and Discussion

**Figure 7:**
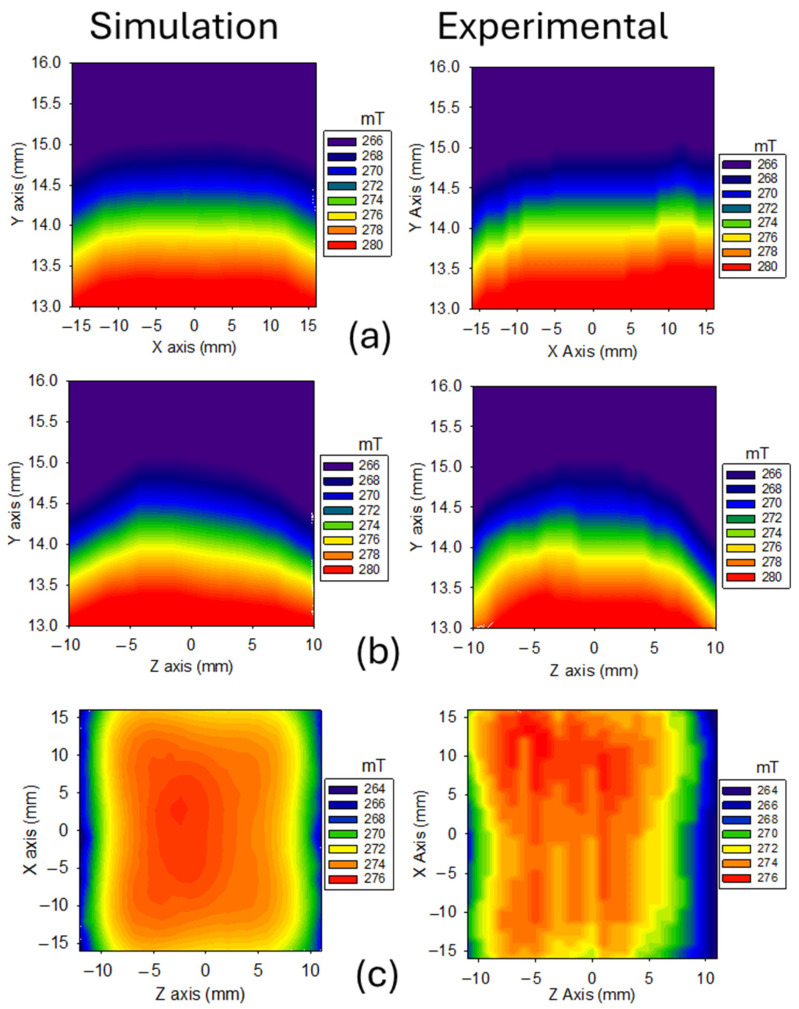
2D plots of the magnetic field generated by the magnet array: (**a**) YX plane, (**b**) ZX plane, and (**c**) ZY plane at a height of 14 mm above the surface of the array.

In Figure 7, the measured magnetic field generated by the unilateral magnet array is compared with the simulated field. A good agreement of the magnetic field was obtained through simulation compared to the measurement of the built sensor. Notably, a relatively uniform zone of the magnetic field B_0_, with an intensity of 274 mT at a height of 14 mm above the magnet surface, is present. This is significant because it ensures the homogeneous zone is inside the samples, allowing for NMR measurements at different depths. Furthermore, this homogeneous zone extends over a rectangular area measuring approximately 14 mm × 30 mm.

**Figure 8:**
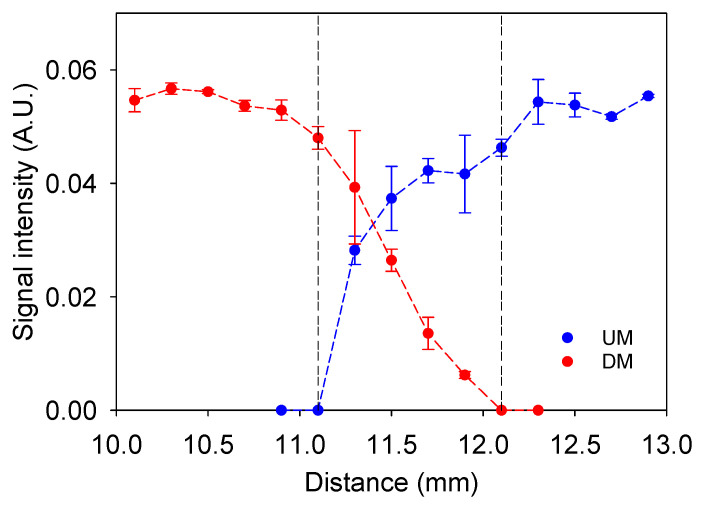
Profiles of signal intensity were obtained, with vertical indicating the thickness of the sensitive zone, approximately 1 mm: upward measurements (UM) and Downward measurements (DM).

Figure 8 displays the signal intensity of an eraser sample 2 mm thick. It was moved parallel to the vertical axis, up and down between the uniform field zone (B_0_). Based on the downward profile (circles and red lines), signal intensity equals zero for the first two measurements because the sample is outside the sensitive zone. However, at 11.7 mm, the NMR signal becomes detectable, and its intensity increases as the sample is moved downward, indicating that most of the sample is entering the sensor’s sensitive zone. The upward profile (circles and blue lines) shows the same trend. The sensitive zone of the sensor spans from 11.1 mm to 12.1 mm, demonstrating that the RF antenna can excite a slice of approximately 1.0 mm thick, which is considered the maximum sensor resolution. To profile different depths in a sample, the sensor must be moved in steps of 1.0 mm.

**Figure 9:**
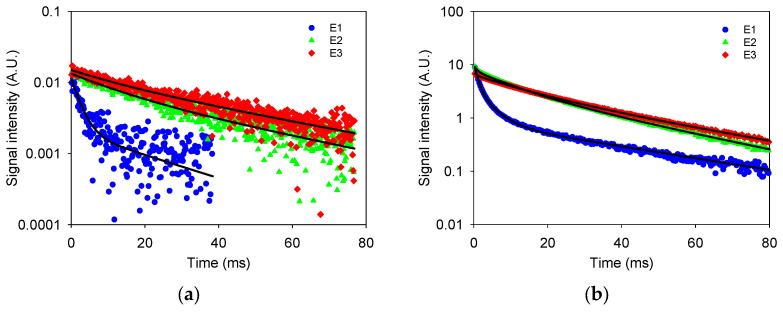
Transverse magnetization decay signals measured in each type of eraser: (**a**) with the constructed unilateral NMR sensor and (**b**) with the Maran DRX system.

Figure 9a shows the CPMG signals obtained from measurements of three different types of erasers. Despite the low signal-to-noise ratio (SNR) of 7, 19, and 22 for E1, E2, and E3, respectively, each material’s signal lifetimes can be observed. This is further supported by measurements taken with the Maran DRX system, as shown in Figure 9b.

**Figure 10:**
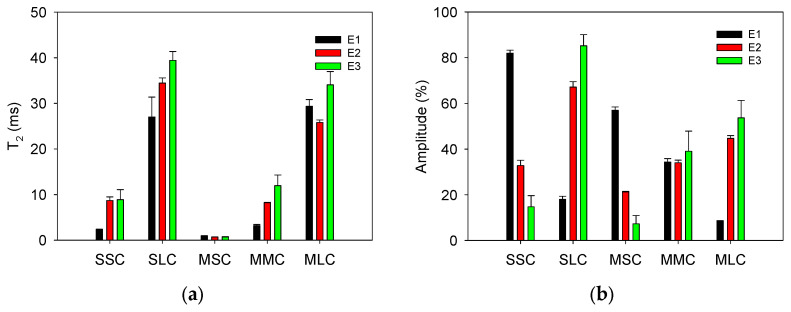
(**a**) T_2_ and (**b**) amplitude values obtained for each eraser. The abbreviations correspond to: Sensor short component (SSC), Sensor long component (SLC), Maran DRX short component (MSC), Maran DRX medium component (MMC), and Maran DRX long component (MLC). The error bars represent ± one standard deviation.

Figure 10 compares the T_2_ (Figure 10a) and amplitude values (Figure 10b) obtained by fitting the data from Figure 9 to exponential decay functions with two and three components. For all erasers, the short (2–10 ms) and long (27–40 ms) T_2_ lifetime components obtained with the sensor were like those obtained with the Maran DRX named medium and long T_2_ components. Intensity and T_2_ time obtained with the sensor showed similar behavior to those obtained with the Maran DRX. However, the shortest component (T_2_ < 1 ms) was undetectable for the sensor because the signal-to-noise ratio of the signals is low compared to the SNR of the signals obtained with the Maran DRX system. A higher quality of the CPMG decays acquired with the Maran system (SNR of 237, 301, and 219 for E1, E2, and E3, respectively) allowed the fitting of signal with a higher number of components, compared to the SNR of the CPMG decays obtained with the sensor (SNR of 7, 19, and 22 for E1, E2, and E3, respectively). This is because, being a unilateral sensor, several factors affect the signal quality; for example, lower B_0_ homogeneity, B_1_ homogeneity, and intensity are dramatically reduced with distance and smaller volume of the excited sample.

**Figure 11:**
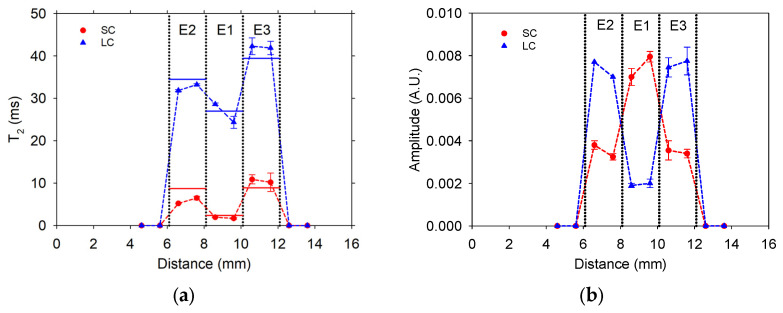
Profiles obtained from specimen measurements composed of slices of three types of erasers: (**a**) T_2_ lifetime and (**b**) signal intensity. The solid horizontal lines represent the measured T_2_ lifetime value for each type of eraser individually.

The signals obtained from different positions of the layered specimen, using slices of three types of erasers, exhibited biexponential decay behavior. The T_2_ lifetime components and their corresponding signal intensities were determined and labeled as short and long T_2_ components and are presented in Figure 11. Differences in T_2_ lifetimes and intensity can be noted between the material type at the position corresponding to the material localization. Notice that the T_2_ lifetime values agree with those measured in each eraser sample individually (horizontal lines in Figure 11a). This demonstrates the ability of the sensor to detect changes due to sample heterogeneity.

**Figure 12:**
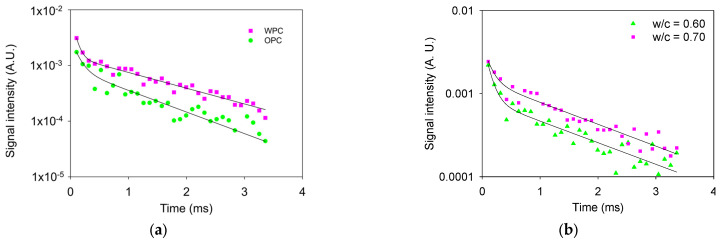
CPMG decays are measured in (**a**) specimens of WPC and OPC pastes with a w/c ratio of 0.60 and (**b**) OPC paste with 0.60 and 0.70 w/c ratios.

Figure 12a presents the CPMG decays in WPC and OPC paste specimens, resulting in an SNR of ≈30 for both signals. These signals were analyzed using a biexponential decay function, which resulted in a short T_2_ lifetime of 0.07 ms (I = 0.073 A.U.) and a long T_2_ lifetime of 1.53 ms (I = 0.0014 A.U.) for WPC paste and 0.11 ms (I = 0.0023 A.U.) and 1.11 ms (I = 0.0009 A.U.) for the OPC paste. The shorter T_2_ values are for water in gel pores, and the longer T_2_ are for water in capillary pores, both present in the hydrated cement paste. The reduction in the long T_2_ lifetime component in the OPC paste compared with WPC, is caused by the iron present in the OPC that enhances the T_2_ relaxation. On the other hand, if the two w/c ratios of OPC pastes are compared (Figure 12b), there is a higher intensity in the 0.70 w/c ratio paste, as expected. The SNR ratios were 26 and 28 for 0.60 and 0.70 w/c pastes, respectively. For the 0.70 w/c ratio, the T_2_ lifetimes were 0.12 ms (I = 0.0026 A.U.) and 1.64 ms (I = 0.0014 A.U.), whereas for the 0.60 w/c ratio, the T_2_ lifetimes were 0.107 ms (I = 0.0037 A.U.) and 1.67 ms (I = 0.0008 A.U.).

**Figure 13:**
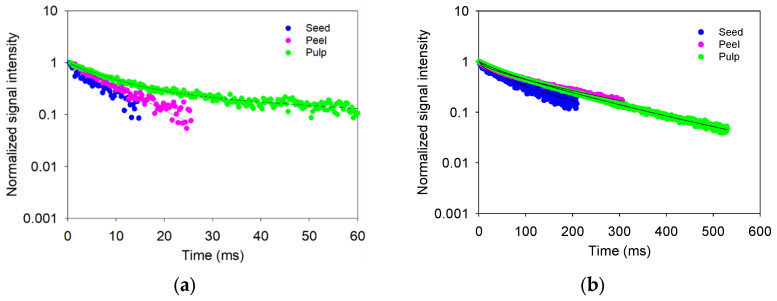
Transverse magnetization decay signals measured from Hass avocado: (**a**) with the built unilateral NMR sensor and (**b**) with the Maran DRX system.

Figure 13 presents the CPMG signal decays measured in the Hass avocado samples. It shows significant differences between the signals obtained for the peel, seed, and pulp, both with the unilateral sensor and with the Maran DRX system.

**Figure 14:**
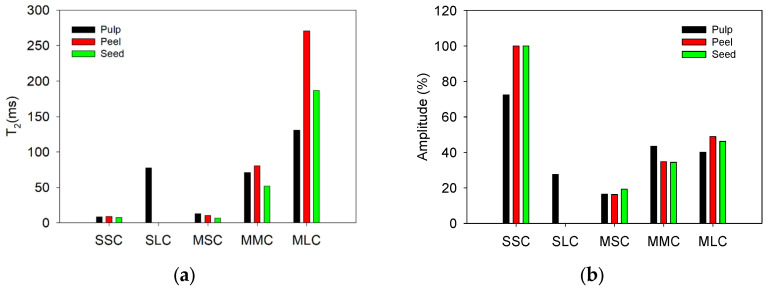
(**a**) T_2_ and (**b**) amplitude values obtained from measurements conducted on a Hass avocado sample.

When analyzing the T_2_ values of the signals (Figure 14), three components were determined with the Maran DRX system, which was expected given that the SNR values obtained were higher than 100 (137 for the seed, 104 for the peel, and 104 for the pulp). This allowed for a better determination of the T_2_ relaxation times. In contrast, with the unilateral sensor, the presence of a B_0_ field gradient across the sensitive zone accelerates the CPMG signal decays. This effect causes a low SNR in the signal (15 for the seed, 25 for the peel, and 27 for the pulp), making resolving three components difficult compared with the Maran DRX system. In this case, only one component was obtained for the seed and peel, while two components were determined for the pulp.

While the Maran DRX system offers a superior quality of the signals, it is limited by sample size and lack of portability. Because of its size, the need to cut the avocado to perform measurements is an explicit limitation, which puts the unilateral sensor at a clear advantage in applications requiring portability and in situ measurements of large samples.

**Figure 15:**
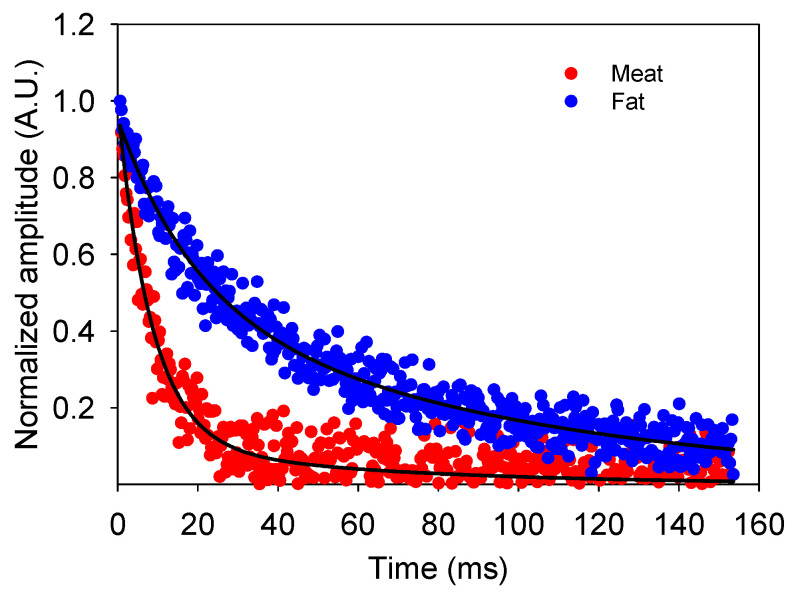
Normalized amplitude decay curves obtained by CPMG measurements on a beef steak performed with the unilateral sensor. The signal corresponding to meat (red) exhibits a faster decay than fat (blue), reflecting each component’s molecular dynamics properties. T_2_ values and associated amplitudes for the fitted components are detailed in the text.

Figure 15 presents the echo train decay measured in a beef steak’s meat (red) and fat (blue) regions using the unilateral sensor. It is evident that the signal corresponding to the meat decays significantly faster compared to the fat, which is reflected in the relaxation times T_2_ and the associated amplitudes. The values obtained for the meat were component 1 (T_2_ = 8.46 ms, 89% of the total amplitude) and component 2 (T_2_ = 59.17 ms, 11% of the total amplitude). For the fat, the results were component 1 (T_2_ = 18.72 ms, 48% of the total amplitude) and component 2 (T_2_ = 90.09 ms, 52% of the total amplitude). In this case, the unilateral sensor demonstrates its ability to distinguish between fat and meat, opening up opportunities for its use in industrial applications, such as food quality control.

## 4. Conclusions

A unilateral ^1^H NMR sensor mounted on a millimetric precision semi-automatic linear displacement mechanism was developed, permitting the measurement of CPMG decay signals in a sensitive volume parallel to the sensor surface. The sensor was used to characterize both organic and inorganic materials. The following conclusions were drawn:

- Simulations of magnetic fields performed using the COMSOL Multiphysics^®^ version 3.4 software resulted yielded similar results to experimentally measured fields. The software facilitated the determination of magnet spacing to generate a relatively homogeneous magnetic field zone. The resulting array yielded a 274 mT zone in a rectangular area measuring approximately 14 mm × 30 mm at 14 mm away from the surface of the magnets.
- Operating at a frequency of 11.71 MHz for ^1^H with a spatial resolution of 1 mm, the sensor was successfully used to acquire NMR signals at different internal depths in both organic and inorganic materials that exhibited relatively short- and long-lived signals. T_2_ lifetime and signal intensity profiles were obtained that showed differences in three types of erasers, as well as differences in signals from white and ordinary Portland cement pastes, in the peel and pulp of Hass avocado samples, and in the fat and meat of a beef steak.

## Figures and Tables

**Table 1:** Parameters of the CPMG sequence used in the measurements with the unilateral sensor and the Maran DRX HF 12/50 instrument.

	Sensor					Maran DRX	
Parameters	Eraser	Cement Paste	Avocado	Meat	Fat	Eraser	Avocado
90° pulse width (μs)	21	21	21	21	21	15.45	15.45
Number of echoes	512	32	512	512	512	512	512
Echo time (μs)	150	105	300	300	300	150	300
Number of scans	16,384	16,384	2048	2048	2048	1024	256
Acquisition time (min)	48	110	56	56	56	4.8	5.2

## Data Availability

The data substantiating the conclusions of this study can be obtained from the corresponding author upon a reasonable request.
